# The impact of intra-abdominal pressure on perioperative outcomes in laparoscopic cholecystectomy: a systematic review and network meta-analysis of randomized controlled trials

**DOI:** 10.1007/s00464-020-07527-2

**Published:** 2020-04-06

**Authors:** Amit D. Raval, Sohan Deshpande, Maria Koufopoulou, Silvia Rabar, Binod Neupane, Ike Iheanacho, Lori D. Bash, Jay Horrow, Thomas Fuchs-Buder

**Affiliations:** 1 Center for Observational and Real-World Evidence, Merck & Co., Inc, Kenilworth, NJ USA; 2 Evidence Synthesis, Modeling, and Communication, Evidera Inc, London, UK; 3 Evidence Synthesis, Modeling, and Communication, Evidera Inc, Montreal, Canada; 4 Merck & Co., Inc., Kenilworth, NJ USA; 5 Department of Anesthesiology & Critical Care, Brabois University Hospital, University de Lorraine, CHRU Nancy, 7 allée du Morvan, 54511 Vandoeuvre-les-Nancy, France

**Keywords:** Cholecystectomy, Laparoscopy, Neuromuscular blockade, Post-operative intra-abdominal pressure, Pneumoperitoneum

## Abstract

**Background:**

Laparoscopic cholecystectomy involves using intra-abdominal pressure (IAP) to facilitate adequate surgical conditions. However, there is no consensus on optimal IAP levels to improve surgical outcomes. Therefore, we conducted a systematic literature review (SLR) to examine outcomes of low, standard, and high IAP among adults undergoing laparoscopic cholecystectomy.

**Methods:**

An electronic database search was performed to identify randomized controlled trials (RCTs) that compared outcomes of low, standard, and high IAP among adults undergoing laparoscopic cholecystectomy. A Bayesian network meta-analysis (NMA) was used to conduct pairwise meta-analyses and indirect treatment comparisons of the levels of IAP assessed across trials.

**Results:**

The SLR and NMA included 22 studies. Compared with standard IAP, on a scale of 0 (no pain at all) to 10 (worst imaginable pain), low IAP was associated with significantly lower overall pain scores at 24 h (mean difference [MD]: − 0.70; 95% credible interval [CrI]: − 1.26, − 0.13) and reduced risk of shoulder pain 24 h (odds ratio [OR] 0.24; 95% CrI 0.12, 0.48) and 72 h post-surgery (OR 0.22; 95% CrI 0.07, 0.65). Hospital stay was shorter with low IAP (MD: − 0.14 days; 95% CrI − 0.30, − 0.01). High IAP was not associated with a significant difference for these outcomes when compared with standard or low IAP. No significant differences were found between the IAP levels regarding need for conversion to open surgery; post-operative acute bleeding, pain at 72 h, nausea, and vomiting; and duration of surgery.

**Conclusions:**

Our study of published trials indicates that using low, as opposed to standard, IAP during laparoscopic cholecystectomy may reduce patients’ post-operative pain, including shoulder pain, and length of hospital stay. Heterogeneity in the pooled estimates and high risk of bias of the included trials suggest the need for high-quality, adequately powered RCTs to confirm these findings.

**Electronic supplementary material:**

The online version of this article (10.1007/s00464-020-07527-2) contains supplementary material, which is available to authorized users.

A standard practice in laparoscopic cholecystectomy is to use carbon dioxide to inflate the peritoneal cavity to form a pneumoperitoneum that creates adequate working space for operating. However, there is no consensus on the optimal intra-abdominal pressure (IAP) for such a pneumoperitoneum, and levels that are too low or high may be associated with key limitations. For instance, very low IAP may restrict the surgeon’s visual field and the space for surgical instruments; conversely, increased IAP could result in cardiopulmonary complications and post-operative pain requiring management with analgesics [1, 2]. Therefore, there is a need for a clear understanding of where to set IAP levels to improve overall surgical outcomes. A key additional consideration is induced muscle paralysis that is often used as part of anesthesia. Specifically, deep neuromuscular block (dNMB) allows for complete relaxation of the abdominal wall musculature and immobilizes the diaphragm, effects that facilitate reduction of the insufflation pressure without compromising the surgical field of vision in laparoscopic abdominal surgeries [3, 4]. However, dNMB has the potential disadvantage of not being reversible by some conventional anti-cholinesterase agents (neostigmine, pyridostigmine), although the introduction of a novel, dNMB-reversing agent to anesthetic practice has allowed more frequent use of the technique [5, 6].

Available evidence to inform practice in this clinical setting includes two systematic literature reviews (SLRs) of randomized controlled trials (RCTs), published in 2013 [7] and 2014 [7, 8]. These reviews suggested that, compared with standard IAP, low IAP pneumoperitoneum for laparoscopic cholecystectomy is associated with significantly reduced post-operative pain (including referred shoulder pain [7]) and analgesic use post-surgery [7, 8]. However, the impact of low IAP on operating times, hospital stays, and other surgical outcomes is not clear. Also, a Cochrane review published around the same time [1] concluded there was no evidence to support using low IAP pneumoperitoneum (defined as < 12 mmHg), compared with standard IAP (defined as 12–16 mmHg), in low-risk surgical patients undergoing planned laparoscopic cholecystectomy. Crucially, no published SLRs have compared the outcomes of low, standard, and high IAP in laparoscopic cholecystectomy in a network meta-analysis (NMA) or used standardized definitions of IAP levels to ensure comparability of data across the studies included in meta-analyses. In light of these data gaps and the availability of additional RCTs beyond those included in existing SLRs on this topic, we conducted an SLR and NMA of RCTs to investigate the impact of different levels of IAP (in clinically well-defined categories of “low,” “standard,” and “high” IAP) on surgical, patient-reported, and resource use outcomes after laparoscopic cholecystectomy.

## Materials and methods

This SLR was conducted in accordance with the quality standards recommended by the Preferred Reporting Items for Systematic Reviews and Meta-Analyses (PRISMA) statement [9] and the Cochrane Handbook for Systematic Reviews [10].

### Criteria for inclusion in the SLR

The SLR included RCTs that assessed surgical, patient-reported, and resource use outcomes associated with using various levels of IAP to produce a pneumoperitoneum in adults undergoing laparoscopic cholecystectomy. Specifically, studies were eligible if they compared at least two of the following levels of IAP: low (< 12 mmHg), standard (12–14 mmHg), and high (≥ 15 mmHg). These IAP categories were chosen on clinical grounds, after examination of the data available in the published literature, and in consultation with expert clinicians. The pre-specified outcomes of interest for consideration in the SLR represented clinically important safety, patient-centered, and efficiency metrics—conversion to open surgery represents a failure of chosen approach, impacting costs and patient comfort; post-operative pain (general and specifically shoulder pain, each at 24 and 72 h post-surgery) affects patient satisfaction and potential safety issues from its treatment; acute bleeding, a safety and cost issue; post-operative nausea or vomiting degrades safety, efficiency, and patient satisfaction; the efficiency metrics of duration of surgery; and length of inpatient stay.

### Database searches

To identify publications evaluating laparoscopic abdominal surgery in adults, the following electronic databases were searched from their dates of inception to September 14, 2018: Embase, MEDLINE, and MEDLINE In-Process via PubMed; the Cochrane Library; the Cochrane Central Register of Controlled Trials (CENTRAL); and the Database of Abstracts of Reviews of Effects (DARE). The searches were not restricted by using terms specific to “cholecystectomy”; this was to capture studies on broader categories of laparoscopic abdominal surgery that might have included separate results for any cholecystectomy subgroups. The searches were limited to English-language publications, studies in humans, and RCTs; in addition, the searches were designed to identify relevant SLRs of RCTs (published since 2016) so that the bibliographies of these reviews could be checked for RCTs not found directly by the database searches. No geographical restrictions were applied in the searches. Full details of the search strings used are provided in Appendix Table 1 in the Supplementary Material.

A gray literature search for relevant RCTs was also conducted in selected conference proceedings from 2017 and 2018 (specifically for the American Society of Anesthesiologists; the European Society of Anesthesiology; and the International Anesthesia Research Society) and registries of ongoing clinical trials (namely, clinicaltrials.gov and World Health Organization International Clinical Trials Registry Platform).

### Study selection and extraction

Two independent researchers screened publications (MK, SR); disagreements were resolved by a third, senior investigator (SD). Pre-specified data of interest on study design, population baseline characteristics, interventions/comparators, and outcomes were extracted from included studies by one researcher (MK or SR), and independently validated by a second researcher (SD). Each included study was assessed using the Cochrane Risk of Bias tool for RCTs [10] and designated as having a low, moderate, or high risk of bias.

### Feasibility assessment of indirect comparison

**Fig. 1:**
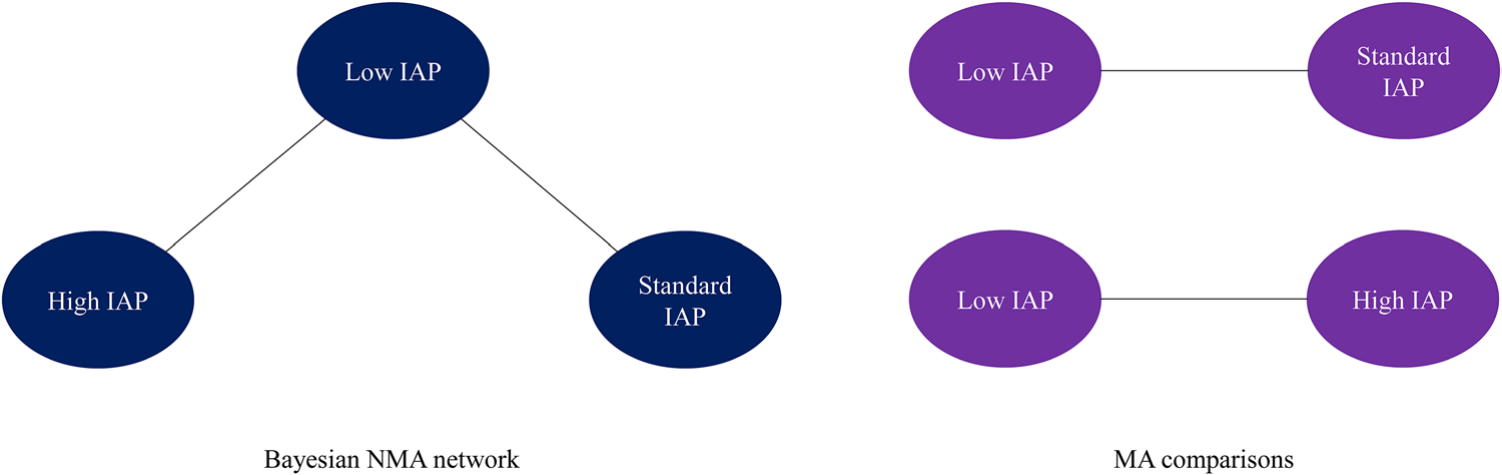
Network diagrams. *IAP* intra-abdominal pressure, *MA* meta-analysis, *NMA* network meta-analysis

We conducted feasibility assessment on the studies identified in the SLR to examine the populations, interventions/comparators, and outcome assessments across the trials. With a view to decrease heterogeneity, analyses were undertaken for trials on patients undergoing laparoscopic cholecystectomy, which also improved transitivity (with respect to disease and surgical procedure). For each possible analysis scenario, all potentially eligible trials were assessed with respect to distributions of possible treatment effect modifiers and covariates to assess whether they were sufficiently similar for a valid indirect comparison; an appropriate decision was then made to include or exclude each trial. After the feasibility assessment, none of the evidence networks had direct and indirect evidence. Evidence was available for comparisons of low vs. high or low vs. standard IAP (Fig. 1). Therefore, disagreement (inconsistency) between direct and indirect evidence was not assessed.

### Statistical analysis

For each outcome of interest, the comparative effects of low, standard, and high IAP were assessed using a Bayesian NMA (when all three IAP levels were included in the evidence network) or Bayesian meta-analysis (MA) (when only two IAP levels were included in the network; i.e., when RCTs provided evidence for only one comparison). Classical/frequentist direct MA for a comparison was also performed as a sensitivity analysis. Where possible, a random-effects (RE) model was used as the primary analytical approach, with a fixed-effect (FE) model used in a sensitivity analysis to assess the impact of heterogeneity on the primary results. However, an FE model was used as for primary analysis if only one study was available for each head-to-head comparison, or where event rates for a binary outcome were very low.

In a Bayesian NMA or MA conducted using the RE model, the prior distribution used for between-study standard deviation (τ) was uniform for a dichotomous (0, 1) outcome and for a continuous outcome (0, b), where “b” was approximately twice the average standard deviation, with mean or median data. In each Bayesian NMA, the first 100,000 samples from Monte Carlo Markov Chain (MCMC) simulation were discarded and another 100,000 samples were saved for posterior inference. Convergence to posterior distribution of each parameter (effects and heterogeneity, where applicable) of interest was achieved in statistical tests and graphical examination. For each comparison, the pooled estimate of a relative effect (e.g., mean difference [MD] for a continuous outcome and odds ratio [OR] for a binary outcome) and corresponding 95% credible intervals (CrI) were obtained to summarize these posterior samples. An MD with the CrI excluding 0 or an OR with CrI excluding 1 was considered statistically significant (from an interpretation perspective). The probability of each treatment being better than any other comparator treatment was also computed as the probability of MD < 0 or log(OR) < 0 for a bad outcome (e.g., length of surgery or conversion to open surgery) and vice versa. The Bayesian NMAs were performed in OpenBUGS (version 3.2.3) via R (version 3.5.1).

For each outcome, the heterogeneity in the estimates of an effect between studies for a pairwise comparison was examined by computing I^2^ (the percentage of between-study heterogeneity beyond chance) [11], estimating between-study variance (τ^2^) using restricted maximal likelihood, and computing the p-value in the Cochran’s Q test of homogeneity. These pairwise meta-analyses were performed using the “*metafor”* package in R (version 3.5.1).

## Results

**Fig. 2:**
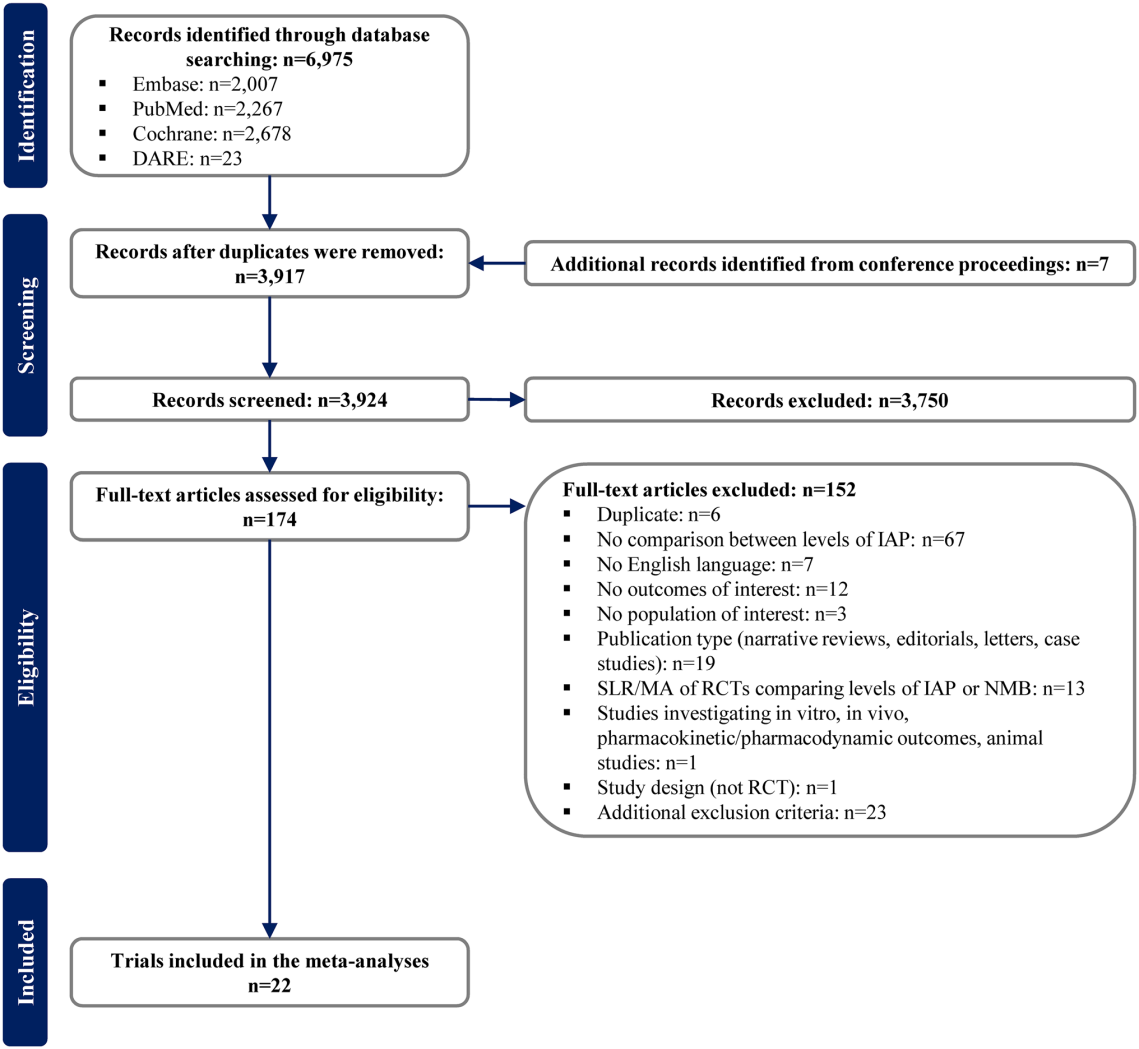
PRISMA diagram. *DARE* Database of Abstracts of Reviews and Effects, *IAP* intra-abdominal pressure, *MA* meta-analysis, *NMB* neuromuscular block, *RCT* randomized controlled trial, *SLR* systematic literature review

The SLR searches generated 3924 unique records, of which 22 were ultimately eligible for and included in the review (Fig. 2). Studies that reported duration of surgery only as a baseline characteristic (*n* = 18) without assessing any post-operative outcomes or presenting extractable data (*n* = 5) were excluded.

Table 1 describes the RCTs included in the SLR. The studies were carried out in Asia (*n* = 14), Europe (*n* = 6), and Africa (*n* = 2), and involved between 20 and 148 patients each. Application of the low, standard, or high IAP categories defined for collation of data across the trials showed that nine studies compared low with standard IAP and four compared low with high IAP. No trial compared standard and high IAP levels. Five studies were single-blinded, six were double-blinded, one was triple-blinded, and six did not report their blinding status. Of the studies that reported blinding details, patients were most frequently blinded (in eight studies), followed by the outcome assessors (in seven studies), and the surgeons (in three studies).

**Table 1:** Characteristics of studies included in the SLR

Author and year	Study arm based on proposed IAP categorization	Study arm (mmHg)	Country	N Randomized	N completed	Who was blinded?
Barczynski (2003) [15]	Low vs. Standard	7 vs. 12	Poland	148	148	P
Bhattacharjee (2017) [29]	Low vs. Standard	9–10 vs. 14	India	80	80	A+P
Celik (2010) [16]	Low vs. Standard	8 vs.12, 14	Turkey	64	60	NR
Chok (2006) [17]	Low vs. Standard	7 vs.12	China	40	40	N
Dexter (1999) [26]	Low vs. High	7 vs. 15	UK	23	20	NR
Ekici (2009) [27]	Low vs. High	7 vs. 15	Turkey	70	52	A
Esmat (2006) [23]	Low vs. High	10 vs. 14	Egypt	109	109	NR
Ibraheim (2006) [30]	Low vs. Standard	6–8 vs. 12–14	NR (assumed Saudi Arabia)	20	20	NR
Joshipura (2009) [18]	Low vs. Standard	8 vs.12	India	26	26	P+S
Kandil (2010) [19]	Low vs. Standard	8, 10 vs. 12, 14	Egypt	100	100	NR
Kanwer (2009) [12]	Low vs. Standard	10 vs. 14	India	60	55	NR
Koc (2005) [20]	Low vs. High	10 vs. 15	Turkey	53	50	A
Ko-iam (2016) [13]	Low vs. Standard	7 vs.14	Thailand	120	115	A+P+S
Meijer (1997) [14]	Low vs. High	5 vs. 15	Netherlands	20	18	NR
Perrakis (2003) [28]	Low vs. High	8 vs. 15	Greece	40	40	P+S
Sandhu (2009) [21]	Low vs. Standard	7 vs. 14	Thailand	140	140	N
Sarli (2000) [24]	Low vs. Standard	9 vs. 13	Italy	94	90	P+A
Singla (2014) [22]	Low vs. Standard	7–8 vs. 12–14	India	100	100	NR
Vijayaraghavan (2014) [31]	Low vs. Standard	8 vs. 12	India	44	43	P+A
Wallace (1997) [37]	Low vs. High	7.5 vs. 15	UK	40	40	P+A
Yasir (2012) [25]	Low vs. Standard	8 vs. 14	India	100	100	NR
Zaman (2015) [38]	Low vs. Standard	7–8 vs. 12–14	India	50	50	NR

*A* assessors of outcomes, *IAP* intra-abdominal pressure, *N* nurse, *NR* not reported, *P* patient, *S* Surgeon, *UK* United Kingdom

**Fig. 3:**
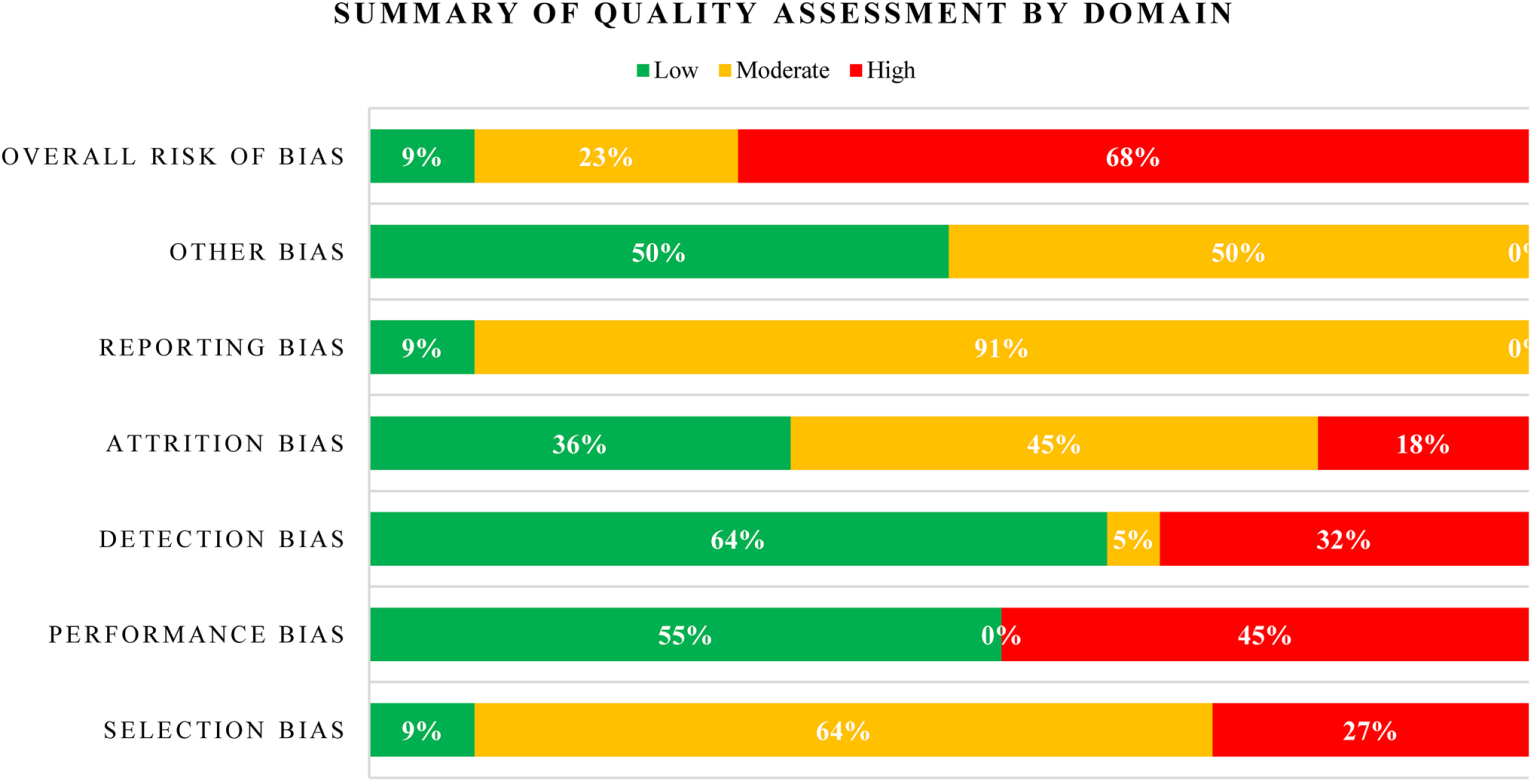
Quality assessment by Cochrane Risk of Bias tool

Table 2 describes the baseline characteristics of the population in each included study. In general, these variables were well balanced between the intervention and comparator groups within the RCTs. The mean age of patients across studies ranged from 35 to 59 years. All trials enrolled patients with an American Society of Anesthesiologists (ASA) status ranging from I to III. Quality assessment of the included studies (using the Cochrane Risk of Bias tool) suggested that 68% had a high risk of bias, 23% a moderate risk, and 9% a low risk (Fig. 3). When the individual bias domains of attrition, detection, performance, and selection were considered, the proportion of the included studies rated as having a high risk of bias ranged from 18 to 45%.

**Table 2:** Baseline characteristics of the included RCTs

Author and year	IAP level	N at baseline	Mean age (SD) [years]	Male (%)	Mean BMI (SD) [kg/m^2^]	ASA physical status classification (%)
Barczynski (2003) [15]	Low	74	48.2 (12.1)	12.2	27.5 (3.2)	I: 70.3 II: 29.7
	Standard	74	47.8 (12.6)	13.5	27.1 (3.3)	I: 63.5 II: 36.5
Bhattacharjee (2017) [29]	Low	40	37.9 (9.3)	NR	24.7 (2.8)	ASA I–II*
	Standard	40	35.3 (11.2)	NR	25.2 (2.6)	ASA I–II*
Celik (2010) [16]	Low	20	42.9 (10.8)	0	NR	ASA I–II*
	Standard	20	43.8 (9.9)	0	NR	ASA I–II*
	Standard	20	45.3 (8.6)	0	NR	ASA I–II*
Chok (2006) [17]	Low	20	47.6 (10.0)	40	NR	I: 95 II: 90
	Standard	20	47.2 (11.0)	40	NR	I: 5 II: 10
Ibraheim (2006) [30]	Low	10	47.2 (6.6)	30	27.0 (1.9)	I: 10 II: 90
	Standard	10	49.9 (10.5)	30	26.9 (2.1)	I: 60 II: 40
Joshipura (2009) [18]	Low	14	57 (NR)	64	27.5 (1.0)	ASA I–II*
	Standard	12	58 (NR)	50	26 (1.4)	ASA I–II*
Kandil (2010) [19]	Low	100	42.4 (10.7) (18–61)	38	NR	ASA I–II*
	Standard	30	NR	NR	NR	NR
Kanwer (2009) [12]	Low	30	NR	NR	NR	NR
	Standard	30	NR	NR	NR	NR
Ko-iam (2016) [13]	Low	60	51.0 (13.3)	18.3	24.6 (4.1)	I: 28.3 II: 71.7
	Standard	60	52.8 (12.1)	30	24.3 (3.4)	I: 41.7 II: 58.3
Sandhu (2009) [21]	Low	70	54 (12.9) (27–78)	12.9	NR	ASA I–II*
	Standard	70	55.2 (13.2) (20–84)	25.7	NR	ASA I–II*
Sarli (2000) [24]	Low	46	Mean 49.3 (22–83)	28.3	NR	ASA I–II*
	Standard	44	Mean 47.7 (27–78)	25	NR	ASA I–II*
Singla (2014) [22]	Low	50	50.6 (14.0)	24	NR	NR
	Standard	50	53.8 (13.8)	40	NR	NR
Vijayaraghavan (2014) [31]	Low	22	44.5 (IQR: 31.5–51.5)	36.4	NR	I: 63.6 II: 66.7
	Standard	21	40 (IQR: 31.5–49.5)	42.9	NR	I: 36.4 II: 33.3
Yasir (2012) [25]	Low	50	NR	NR	NR	NR
	Standard	50	NR	NR	NR	NR
Zaman (2015) [38]	Low	25	NR	NR	NR	ASA I–II*
	Standard	25	NR	NR	NR	ASA I–II*
Dexter (1999) [26]	Low	10	Mean: 48 (range: 19–72)	30	Mean: 25.4 (range: 18.1–32.2)	ASA I–II*
	High	10	Mean: 56 (range: 27–71)	40	Mean: 27 (range: 20.1–30.9)	ASA I–II*
Ekici (2009) [27]	Low	20	52.2 (10.1)	10	28.5 (4.8)	ASA I–II*
	High	32	49.3 (12.6)	18.8	28.4 (5.1)	ASA I–II*
Esmat (2006) [23]	Low	37	47.8 (NR)	27	NR	ASA I–II*
	Low	38	45.8 (NR)	26.3	NR	ASA I–II*
	High	34	46.6 (NR)	26.5	NR	ASA I–II*
Koc (2005) [20]	Low	25	46.3 (15.5)	12	NR	ASA I–III*
	High	25	47.9 (15.2)	24	NR	ASA I–III*
Meijer (1997) [14]	Low	9	(22–50)	11	NR	ASA I–II*
	High	9	(30–52)	22	NR	ASA I–II*
Perrakis (2003) [28]	Low	20	58.5 (33–79)	35	26.4 (21.2–34.3)	I: 60 II: 40
	High	20	55 (30–79)	15	25.3 (19.8–43.6)	I: 65 II: 35
Wallace (1997) [37]	Low	20	Median: 59 (IQR: 52–64)	30	Median: 26.4 (IQR: 24.8–28.4)	NR
	High	20	Median: 56 (IQR: 50–64)	20	Median: 25.9 (IQR: 23.1–29.5)	NR

^*^Specific % is not reported

*ASA* American Society of Anesthesiologists, *BMI* body mass index, *IAP* intra-abdominal pressure, *IQR* interquartile range, *NR* not reported, *SD* standard deviation

### Surgical outcomes

**Fig. 4:**
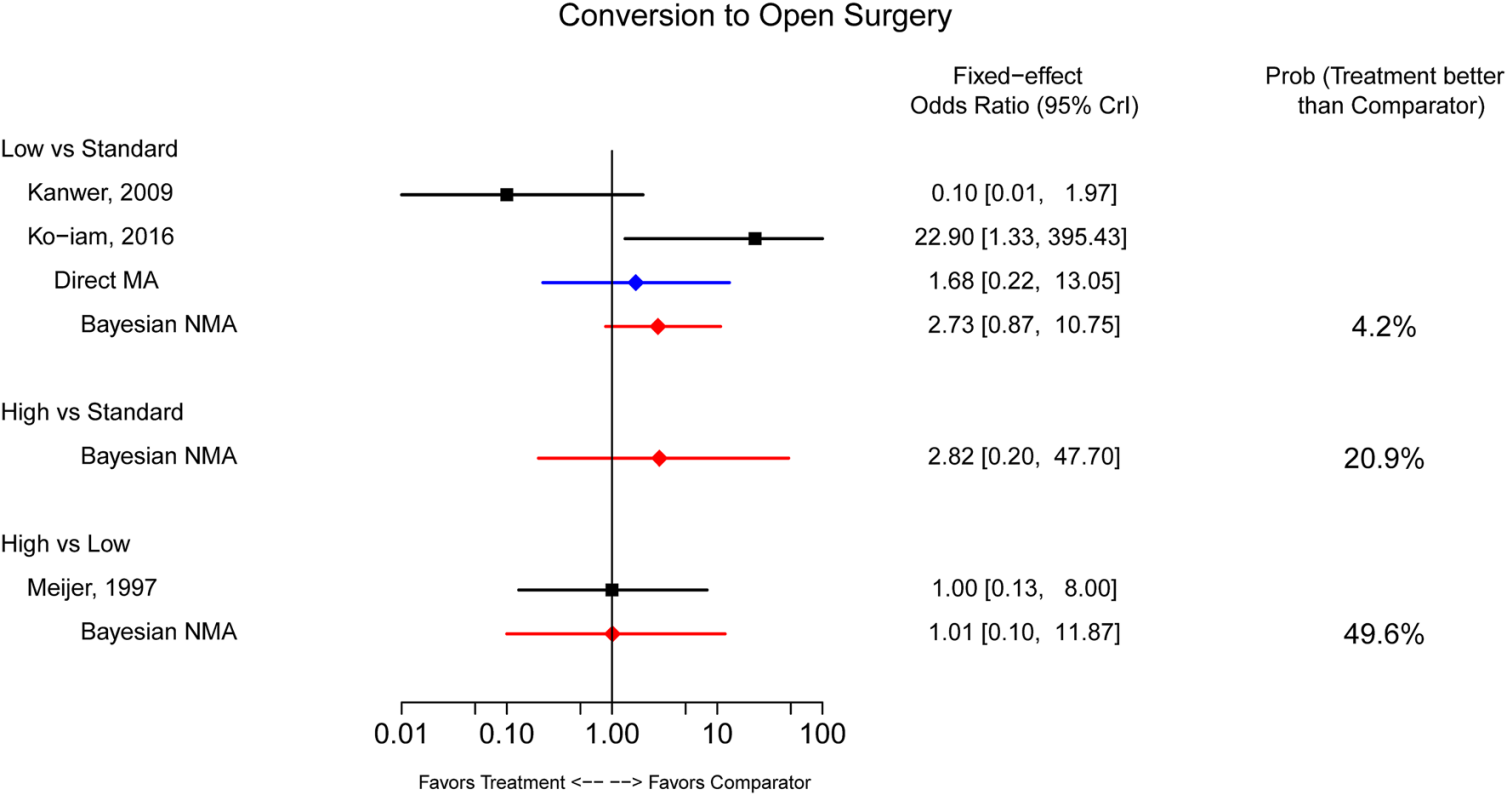
Surgical conditions outcomes. *CrI* credible interval, *MA* meta-analysis, *NMA* network meta-analysis, *Prob* probability

Three studies [12–14] that reported on the need to convert laparoscopic surgery to open surgery, were included in a Bayesian NMA of this outcome. The analysis (conducted using an FE model because of very low rates of the event of interest) showed no statistically significant differences between the three IAP levels with respect to then need to convert to open surgery (Fig. 4).

### Patient-reported outcomes

To assess post-operative pain, most of the included RCTs used a visual analog scale ranging from 0 to 10, where 0 indicated “no pain at all” and 10 “worst imaginable pain.” For studies that used a scale from 0 to 100, we converted the data to a 0 to 10 scale before they were included in the meta-analyses.

**Fig. 5:**
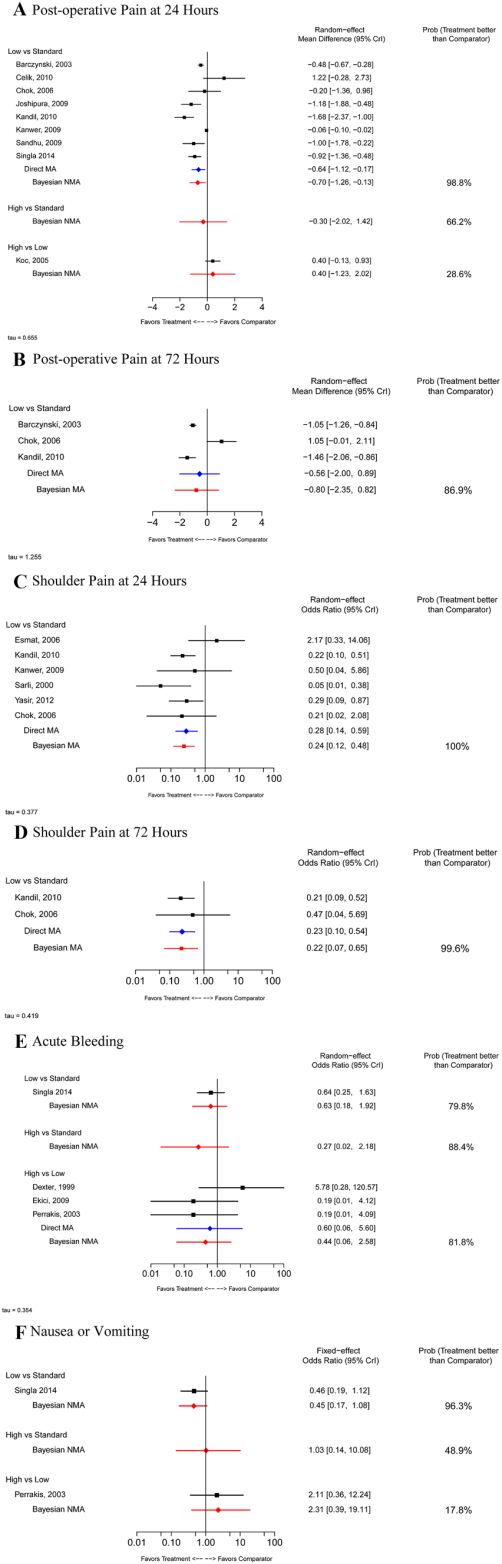
Patient outcomes. *CrI* credible interval, *MA* meta-analysis, *NMA* network meta-analysis, *Prob* probability

Nine studies [12, 15–22] that reported on post-operative pain 24 h post-surgery were included in the Bayesian NMA (Fig. 5A). The results of this analysis showed that when compared with standard IAP, low IAP was associated with significantly lower pain scores at 24 h (MD: − 0.70; 95% CrI − 1.26, − 0.13, *n* = 8 studies). There was no significant difference between low and high IAP with regard to this outcome (*n* = 1 study). No studies directly compared standard and high IAP, but a Bayesian NMA showed a non-statistically significant difference between the levels (high between-study heterogeneity: *I*^2^ = 93.3%).

Three studies [15, 17, 19] reported on post-operative pain 72 h post-surgery and were included in a Bayesian MA (Fig. 5B). The analysis suggested no significant difference between low and standard IAP for the severity of this outcome, with high between-study heterogeneity in frequentist meta-analysis (*I*^2^ = 94.9%).

Six studies [12, 17, 19, 23–25] reported on the dichotomous outcome of post-operative shoulder pain 24 h after laparoscopic cholecystectomy (Fig. 5C). A Bayesian MA showed that, compared with standard IAP, low IAP was associated with a statistically significantly reduced risk of shoulder pain (OR 0.24; 95% CrI 0.12, 0.48) (*I*^2^ = 89.9%). Two studies also reported on shoulder pain 72 h after surgery [17, 19] (Fig. 5D) and were included in a Bayesian MA. The analysis showed that, compared with standard IAP, low IAP was associated with a statistically significantly reduced risk of such pain (OR 0.22; 95% CrI 0.07, 0.65).

Four studies [22, 26–28] reported on acute post-operative bleeding (Fig. 5E) and were included in a Bayesian NMA. No difference was observed among the IAP levels with respect to this outcome. Only two studies [22, 28] reported on post-operative nausea and vomiting (Fig. 5F). One study compared low to standard IAP; the other compared low to high IAP. The FE analysis of these limited data suggested no difference in the likelihood of post-operative nausea and vomiting among the three levels of IAP.

### Healthcare resource utilization

**Fig. 6:**
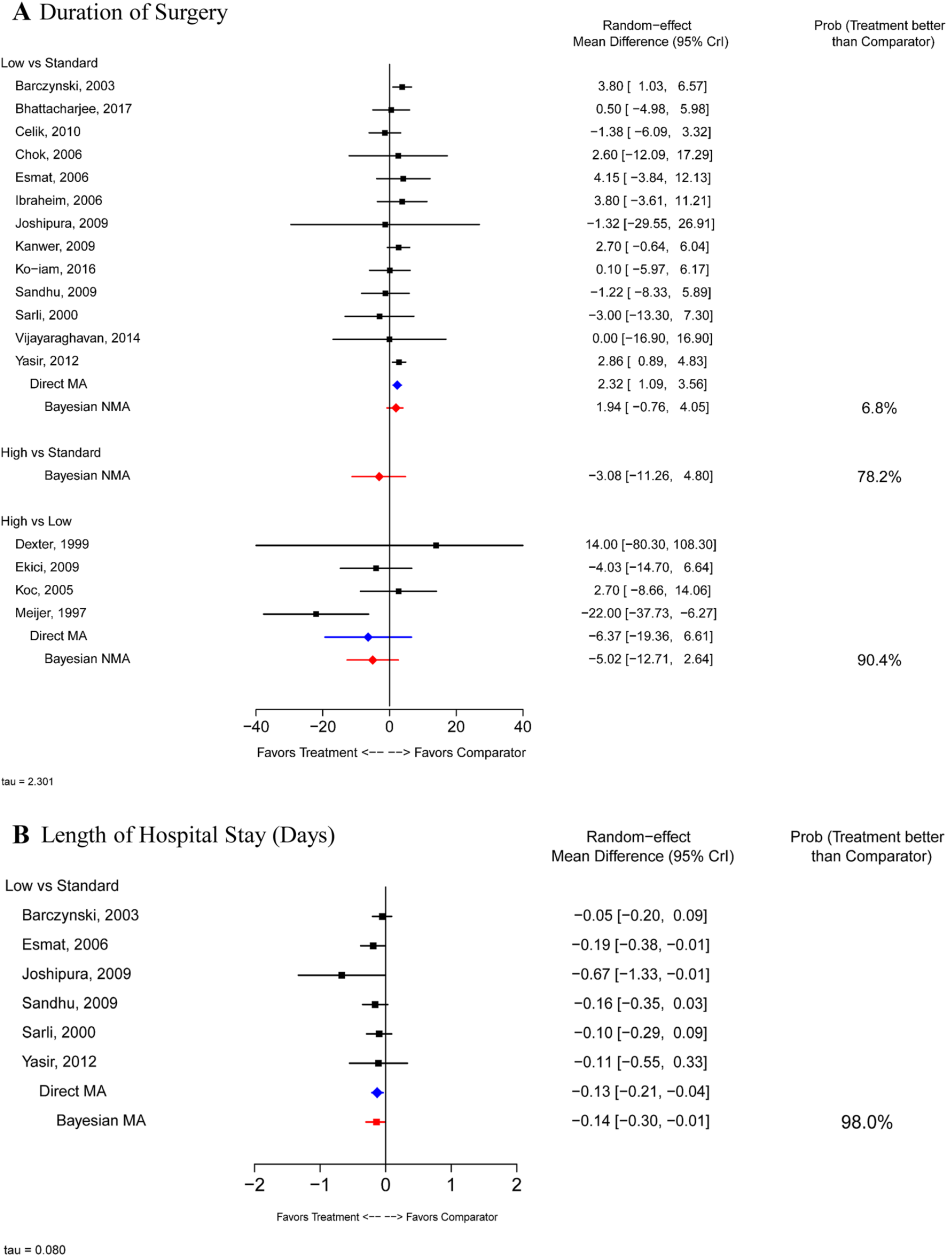
Healthcare resource utilization. *CrI* credible interval, *MA* meta-analysis, *NMA* network meta-analysis, *Prob* probability

Seventeen RCTs [12–18, 20, 21, 23–27, 29–31] were included in the NMA that assessed duration of surgery (Fig. 6A). There was considerable heterogeneity across the studies and, overall, no significant difference between IAP levels was observed in the RE analysis. Six studies [15, 18, 21, 23–25] reported on length of inpatient stay (days) (Fig. 6B). Bayesian NMA suggested that low IAP resulted in a shorter stay (MD − 0.14 days; 95% CrI − 0.30, − 0.01) compared with standard IAP. No studies reported comparisons between the length of stay associated with high IAP vs. low or standard IAP.

Tabular findings on NMA rank probabilities/league tables for each study outcome are provided in the Appendix Table 2 to 11 in the Supplementary Material, with the treatment ranking for further information.

## Discussion

We conducted an SLR and NMA to compare the effects of three potential levels of IAP used laparoscopic cholecystectomy—namely, low (< 12 mmHg), standard (12–14 mmHg), and high (≥ 15 mmHg)—on surgical and patient-reported outcomes and healthcare resource utilization. The SLR included 22 studies.

Our findings suggest that, compared with standard IAP, low IAP was associated with significant reductions in post-operative pain 24 h post-surgery and in shoulder pain specifically 24 and 72 h post-surgery. Only one study compared post-operative pain at 24 h in low vs. high IAP—it showed no statistical difference between the levels. While there were no head-to-head RCT data available on the comparative effects of standard IAP and high IAP with respect to post-operative pain at 24 h, an indirect comparison through a Bayesian NMA suggested there was no statistical difference between the levels, although high IAP had a numerical advantage. There was high heterogeneity (I^2^ ≥ 90%) for the comparison of low with standard IAP; the results of this direct analysis should be interpreted with caution. The same caution should be applied to the indirect result for the comparison between standard and high IAP. Interestingly, no studies directly compared standard with high IAP levels for any of the nine outcomes of interest.

Our analysis found that, compared with standard IAP, low IAP was associated with a statistically significant, but modest, reduction in the length of inpatient stay. However, for the other outcomes of duration of surgery, conversion to open surgery, acute bleeding, and nausea/vomiting, there were no statistically significant differences between low, standard, and high IAP levels. The data available were limited, or the event rates were low for these outcomes. Therefore, the power to detect significant differences was low, even if IAP levels had a major effect on any of these outcomes.

Our findings on the relative outcomes with low and standard IAP were broadly consistent with those cited in prior SLR reports. The 2013 narrative synthesis by Donatsky et al. [7] (which did not include quantitative analysis) concluded that low IAP in laparoscopic cholecystectomy was associated with lower post-operative shoulder pain at 24 h compared with standard IAP. In 2014, Hua et al. [8] concluded that low IAP was associated with a significant reduction in post-operative pain compared with standard IAP. However, the inclusion criteria and classification of IAP levels differed between the Hua et al. review [8] and ours. Specifically, while we excluded RCTs that reported duration of surgery only at baseline rather than as a truly comparative surgical outcome and did not report any other relevant outcomes, Hua et al. included such studies. Also, Hua et al. compared only two levels of IAP (low: 7–10 mmHg and standard: 12–15 mmHg), while our study compared three levels through an NMA. In the review by Donatsky et al. [7], a standardized classification of IAP levels was not adopted; instead, the authors narratively summarized the results based on the IAP levels reported in each included study.

In their MA comparing low (< 12 mmHg) with standard (12–16 mmHg) IAP in laparoscopic cholecystectomy, Gurusamy et al. found no meaningful differences between the two in duration of surgery, length of stay, mortality, serious adverse events, or conversion to open surgery [1]. While our analysis identified no clear differences in duration of surgery, surgical complications, or conversion to open surgery, it did indicate a reduced length of inpatient stay with low IAP compared with standard IAP.

In a study in patients undergoing laparoscopic surgery, Matsuzaki et al. [32] found that low IAP (8 mmHg), compared with standard IAP (12 mmHg) had lower adverse outcomes in molecular levels (e.g., when comparing the expression of the genes that encode inflammatory cytokine signaling molecules). Biologically, animal experiments have indicated that high IAP might have higher adverse impact (e.g., higher rates of peritoneal tissue hypoxia and peritoneal dissemination) [33–36]. This molecular and experimental evidence supports the use of low IAP to improve perioperative outcomes.

Some inconsistencies between findings across RCTs in the literature are due in part to the lack of standardized IAP categories and the heterogeneity of contributing evidence. Therefore, in attempt to improve upon existing data, we used methodology more likely to provide robust and generalizable data. First, our review was based only on evidence from RCTs. Second, we considered only studies that included data specifically on cholecystectomy to improve homogeneity and comparability. Third, IAP was classified into the three pre-specified levels in a clinically meaningful way.

Our review has some limitations. For example, there was substantial heterogeneity among comparisons of low and standard IAP for post-operative pain (I^2^ ≥ 90%). This may have been due to variability in patient characteristics of age, body mass index, or ASA physical status classification, details of which often were not reported across studies. Another limitation was that the studies included patients of Asian/Middle Eastern, European, and African origins, and were conducted in 11 countries. This geographic, ethnic, and cultural diversity could mean that pain and other outcomes might have been perceived differently or that the scales used to report certain outcomes might have been understood differently from study to study. Variation in surgeons’ skills and practices, hospital protocols, or hospital resources (e.g., quality of equipment, availability of nurses and other staff during surgery and recovery) could also have contributed to this observed heterogeneity. In particular, length of hospital stay after surgery is likely to have varied with the economies and healthcare systems of the different countries. It is also important to note that we could not assess any inconsistencies between direct and indirect evidence because none of the comparisons had both types available to allow such an evaluation. Lastly, an SLR/NMA is only as robust as the studies contributing to it. This is highly pertinent in our analysis, since most of the included studies had a high risk of bias per the Cochrane Risk of Bias Tool—some study samples were small; the results in all relevant outcomes were not reported consistently; and bias due to selective reporting cannot be ruled out. Given that we analyzed nine outcome scenarios, presenting the certainty of evidence (confidence or robustness of each estimate) for each relative effect of IAP level per each outcome using the Grading of Recommendations Assessment, Development, and Evaluation tool was not feasible. We feel that we have highlighted all concerns and inconsistencies associated with the results from our analyses in detail under the limitations of this SLR.

## Conclusion

Compared with standard IAP, using a low IAP pneumoperitoneum during laparoscopic cholecystectomy may reduce patients’ post-operative pain (including shoulder pain) and the length of hospital stay. Our findings are consistent with the existing literature. Therefore, low IAP may be preferred in the clinical practice. However, the data should be interpreted with caution given the high chance of bias and high level of heterogeneity, especially for the post-operative pain outcome. Our review highlights the need for robustly designed and executed, adequately powered RCTs to confirm the findings presented.

## Electronic supplementary material

Below is the link to the electronic supplementary material.

Supplementary file1 (DOCX 58 kb)
