# Supplementary material for: The impact of intra-abdominal pressure on perioperative outcomes in laparoscopic cholecystectomy: a systematic review and network meta-analysis of randomized controlled trials
Source: Surg Endosc. 2020 Apr 6;34(7):2878–90. doi: 10.1007/s00464-020-07527-2 (PMC7270984; doi:10.1007/s00464-020-07527-2)
Supplement: Supplementary file 1 — Supplementary file1 (DOCX 58 kb) [file 464_2020_7527_MOESM1_ESM.docx]

# Supplemental Material

## Search Strategies

### Appendix Table 1. Search Strategy

| Search Number | Search Terms | Number of Hits |
| --- | --- | --- |
| **EMBASE Search Strategy** | | |
| #1 | 'neuromuscular block*':ti,ab OR ((neuromuscul* NEXT/3 block*):ti,ab) OR 'neuromuscular blocking agent' OR 'neuromuscular blocking' | 19,870 |
| #2 | surgery:ti,ab OR surgical:ti,ab OR operation*:ti,ab OR 'surgical procedure*':ti,ab OR 'surgical condition*':ti,ab OR 'surgery'/exp | 5,328,315 |
| #3 | #1 AND #2 | 8,015 |
| #4 | 'pneumoperitoneum'/exp OR pneumoperitoneum:ti,ab OR 'insufflation'/exp OR insufflation:ti,ab OR 'intraabdominal pressure':ti,ab OR 'intra-abdominal pressure':ti,ab OR 'intra abdominal pressure':ti,ab | 31,009 |
| #5 | 'laparoscopy'/exp OR ((laparoscop*:ti,ab OR 'robot*':ti,ab) AND (surgery:ti,ab OR operation*:ti,ab OR 'surgical procedure*':ti,ab OR 'surgery'/exp)) OR 'minimally invasive surgery':ti,ab OR 'endoscopy'/exp OR endoscopy:ti,ab | 660,250 |
| #6 | #4 AND #5 | 11,269 |
| #7 | #3 OR #6 | 19,140 |
| #8 | 'randomized controlled trial'/exp OR 'randomized controlled trial'/exp/mj OR rct:ti,ab OR ((('randomized' OR 'randomised') NEXT/2 trial):ti,ab) OR (doubl* AND blind*:ti,ab) OR (singl* AND blind*:ti,ab) OR ('open':ab,ti AND label*:ti,ab) OR 'clinical trial':ti,ab | 856,344 |
| #9 | 'meta analysis'/exp OR 'systematic review (topic)'/exp OR 'meta analysis (topic)'/exp OR 'systematic review'/exp OR 'systematic and review' OR 'meta AND analysis' | 296,875 |
| #10 | #8 OR #9 | 1,118,144 |
| #11 | comment*:it OR editorial:it OR letter:it OR 'case report'/exp OR 'case study':ti,ab,de OR 'case studies':ti,ab,de | 3,851,882 |
| #12 | #7 AND #10 | 2,391 |
| #13 | #12 NOT #11 AND [humans]/lim AND [english]/lim | 2,007 |
| **PubMed Search Strategy** | | |
| #1 | neuromuscular block*[tiab] OR (neuromuscul*[tiab] AND block* [tiab]) OR Neuromuscular Blockade[MESH] OR “Neuromuscular Blocking Agents”[MESH] OR “neuromuscular blocking agent” OR “neuromuscular blocking” | 17,970 |
| #2 | surgery[tiab] OR surgical[tiab] OR operation*[tiab] OR surgical procedure*[tiab] OR surgical condition*[tiab] OR “Surgical Procedures, Operative”[MESH] | 3,901,483 |
| #3 | Search #1 AND #2 | 5,409 |
| #4 | (“Pneumoperitoneum, Artificial”[MESH] OR “Pneumoperitoneum”[MESH] OR pneumoperitoneum[tiab] OR “insufflation”[MESH] OR insufflation[tiab] OR “intraabdominal pressure”[tiab] OR “intra-abdominal pressure”[tiab] OR “intra abdominal pressure”[tiab]) | 17,435 |
| #5 | laparoscopy[MESH] OR laparoscopy[tiab] OR ((laparoscop*[tiab] OR robot*[tiab]) AND (surgery[tiab] OR operation*[tiab] OR surgical procedure*[tiab] OR “Surgical Procedures, Operative”[MESH])) OR “minimally invasive surgery”[tiab] OR “endoscopy”[MESH] OR  endoscopy[tiab] | 389,529 |
| #6 | #4 AND #5 | 6,973 |
| #7 | #3 OR #6 | 12,313 |
| #8 | “Randomized Controlled Trial” [Publication Type] OR “Randomized Controlled Trials as Topic” [Mesh] OR “randomized controlled trial”[tiab] OR “randomised controlled trial”[tiab] OR rct[tiab] OR ((randomized[tiab] OR randomised[tiab] OR random*[tiab]) AND trial* [tiab]) OR “clinical trial”[tiab] OR “Clinical Trial” [Publication Type] OR (doubl*[tiab] AND blind*[tiab]) OR (singl*[tiab] AND blind*[tiab]) OR (“open”[tiab] AND label*[tiab]) | 1,142,138 |
| #9 | (meta-analysis[Publication Type] OR "Meta-Analysis as Topic" [Mesh] OR meta analy*[tiab] OR (meta AND analysis) OR systematic review*[tiab] OR (systematic AND review)) | 386,572 |
| #10 | #8 OR #9 | 1,447,054 |
| #11 | comment [Publication Type] OR editorial [Publication Type] OR letter[Publication Type] OR Case Reports[Publication Type] | 3,352,651 |
| #12 | #7 AND #10 | 2,759 |
| #13 | #12 NOT #11 | 2,717 |
| #14 | #12 NOT #11 Filters: Humans | 2,520 |
| #15 | #12 NOT #11 Filters: Humans; English | 2,267 |
| **Cochrane Library Search Strategy** | | |
| #1 | (“neuromuscular block*”:ti,ab,kw OR (neuromuscul*:ti,ab,kw NEXT/3 block*:ti,ab,kw) OR “neuromuscular blocking” OR “neuromuscular blocking agent”) | 2,719 |
| #2 | MeSH descriptor: [Neuromuscular Blockade] explode all trees | 428 |
| #3 | MeSH descriptor: [Neuromuscular Blocking Agents] explode all trees | 1,367 |
| #4 | #1 OR #2 OR #3 | 3,162 |
| #5 | ((surgery:ti,ab,kw OR surgical:ti,ab,kw OR operation*:ti,ab,kw OR "surgical procedure*":ti,ab,kw OR "surgical condition*":ti,ab,kw)) | 182,217 |
| #6 | MeSH descriptor: [Surgical Procedures, Operative] explode all trees | 108,670 |
| #7 | #5 OR #6 | 221,552 |
| #8 | #4 AND #7 | 1,964 |
| #9 | ((pneumoperitoneum:ti,ab,kw OR insufflation:ti,ab,kw OR “intraabdominal pressure”:ti,ab,kw OR “intra-abdominal pressure”:ti,ab,kw OR “intra abdominal pressure”:ti,ab,kw)) | 2,074 |
| #10 | MeSH descriptor: [Pneumoperitoneum, Artificial] explode all trees | 279 |
| #11 | MeSH descriptor: [Pneumoperitoneum] explode all trees | 65 |
| #12 | MeSH descriptor: [Insufflation] explode all trees | 264 |
| #13 | #9 OR #10 OR #11 OR #12 | 2,074 |
| #14 | laparoscopy:ti,ab,kw OR ((laparoscop*:ti,ab,kw OR robot*:ti,ab,kw) AND (surgery:ti,ab,kw OR operation*:ti,ab,kw OR “surgical procedure*”:ti,ab,kw)) OR “minimally invasive surgery”:ti,ab,kw OR endoscopy:ti,ab,kw | 22,294 |
| #15 | MeSH descriptor: [Laparoscopy] explode all trees | 5,057 |
| #16 | (((laparoscop*:ti,ab,kw OR robot*:ti,ab,kw))) | 16,195 |
| #17 | MeSH descriptor: [Surgical Procedures, Operative] explode all trees | 108,670 |
| #18 | MeSH descriptor: [Endoscopy] explode all trees | 15,748 |
| #19 | #14 OR #15 OR (#16 AND #17) OR #18 | 29,554 |
| #20 | #13 AND #19 | 1,231 |
| #21 | #8 OR #20 | 3,118 |
| #22 | MeSH descriptor: [Randomized Controlled Trial] explode all trees | 137 |
| #23 | MeSH descriptor: [Randomized Controlled Trials as Topic] explode all trees | 13,527 |
| #24 | (meta-analysis:pt OR meta analy*:ti,ab,kw OR (meta AND analysis) OR systematic review*:ti,ab,kw) | 22,397 |
| #25 | “randomized controlled trial” OR “randomised controlled trial” OR rct OR ((randomized OR randomised OR random*) NEXT/3 trial*) OR “clinical trial” OR (doubl* AND blind*) OR (singl* AND blind*) OR (open AND label*) | 943,784 |
| #26 | MeSH descriptor: [Meta-Analysis] explode all trees | 0 |
| #27 | #22 OR #23 OR #24 OR #25 OR #26 | 945,368 |
| #28 | (comment OR editorial OR letter OR "case report*"):pt | 9,222 |
| #29 | #21 AND #27 | 2,694 |
| #30 | #29 NOT #28 | 2,678 |
| **DARE Search Strategy** | | |
| #1 | (neuromuscular block*) IN DARE | 29 |
| #2 | (neuromuscul* AND block*) IN DARE | 39 |
| #3 | (neuromuscular blocking agent) IN DARE | 3 |
| #4 | (neuromuscular blocking) IN DARE | 16 |
| #5 | MeSH DESCRIPTOR Neuromuscular Blockade EXPLODE 1 IN DARE | 9 |
| #6 | MeSH DESCRIPTOR Neuromuscular Blocking Agents EXPLODE 1 IN DARE | 6 |
| #7 | (surgery) IN DARE | 8,054 |
| #8 | (surgical) IN DARE | 4,684 |
| #9 | (operation*) IN DARE | 704 |
| #10 | (surgical procedure) IN DARE | 146 |
| #11 | (surgical condition) IN DARE | 0 |
| #12 | MeSH DESCRIPTOR Surgical Procedures, Operative EXPLODE 1 IN DARE | 139 |
| #13 | #1 OR #2 OR #3 OR #4 OR #5 OR #6 | 39 |
| #14 | #7 OR #8 OR #9 OR #10 OR #11 OR #12 | 9,633 |
| #15 | #13 AND #14 | 6 |
| #16 | MeSH DESCRIPTOR Pneumoperitoneum EXPLODE 1 IN DARE | 2 |
| #17 | (pneumoperitoneum) IN DARE | 17 |
| #18 | MeSH DESCRIPTOR insufflation EXPLODE 1 IN DARE | 19 |
| #19 | (insufflation) IN DARE | 47 |
| #20 | (intraabdominal pressure) IN DARE | 0 |
| #21 | (intra-abdominal pressure) IN DARE | 3 |
| #22 | (intra abdominal pressure) IN DARE | 3 |
| #23 | MeSH DESCRIPTOR laparoscopy EXPLODE 1 2 IN DARE | 623 |
| #24 | (laparoscopy) IN DARE | 718 |
| #25 | (minimally invasive surgery) IN DARE | 32 |
| #26 | (endoscopy) IN DARE | 582 |
| #27 | MeSH DESCRIPTOR endoscopy EXPLODE 1 2 IN DARE | 140 |
| #28 | (robot* AND surgery) IN DARE | 115 |
| #29 | (robot* AND procedure) IN DARE | 13 |
| #30 | (robot* AND operation) IN DARE | 14 |
| #31 | #16 OR #17 OR #18 OR #19 OR #20 OR #21 OR #22 | 60 |
| #32 | #23 OR #24 OR #25 OR #26 OR #27 OR #28 OR #29 OR #30 | 1,263 |
| #33 | #31 AND #32 | 17 |
| #34 | #15 OR #33 | 23 |

Abbreviations: DARE = Database of Abstracts of Reviews and Effects; MeSH = medical subject heading

## Rank Probabilities and League Tables

### Appendix Table 2. Conversion to Open Surgery: Rank Probabilities and SUCRA of Each Treatment (%)

| **Treatment** | **Rank 1** | **Rank 2** | **Rank 3** | **SUCRA** |
| --- | --- | --- | --- | --- |
| Standard | 77.3 | 20.3 | 2.5 | 87.4 |
| High | 20.6 | 29.4 | 50.0 | 35.3 |
| Low | 2.1 | 50.4 | 47.5 | 27.3 |

Lower value of the estimate was considered better while ranking treatments.

Abbreviation: SUCRA = surface under the cumulative ranking

### Appendix Table 3. Conversion to Open Surgery: Estimates and 95% CrIs of OR and Probabilities for Each Comparison

| **Intervention** | **Comparator Standard**  **OR [95% CrI] %** | **Low**  **OR [95% CrI] %** |
| --- | --- | --- |
| Low | 2.73 [0.87, 10.75] 4.2% |  |
| High | 2.82 [0.2, 47.7] 20.9% | 1.01 [0.1, 11.87] 49.6% |

The estimates and the probabilities (%) are for the Interventions (in first column) vs. Comparators (in the second, third, etc. columns).

Probability (%) for the comparison of two specific treatments (not to be confused with the rank probability) indicates the chance that an intervention is better than the comparator treatment.

Abbreviations: CrI = credible interval; OR = odds ratio

### Appendix Table 4. Post-operative Pain at 24 Hours: Rank Probabilities and SUCRA of Each Treatment (%)

| **Treatment** | **Rank 1** | **Rank 2** | **Rank 3** | **SUCRA** |
| --- | --- | --- | --- | --- |
| Low | 70.6 | 28.9 | 0.4 | 85.1 |
| High | 28.6 | 37.7 | 33.8 | 47.4 |
| Standard | 0.8 | 33.4 | 65.8 | 17.5 |

Lower value of the estimate was considered better while ranking treatments.

Abbreviation: SUCRA = surface under the cumulative ranking

### Appendix Table 5. Post-operative Pain at 24 Hours: Estimates and 95% CrIs of MD and Probabilities for Each Comparison

| **Intervention** | **Comparator Standard**  **MD [95% CrI] %** | **Low**  **MD [95% CrI] %** |
| --- | --- | --- |
| Low | -0.7 [-1.26, -0.13] 98.8% |  |
| High | -0.3 [-2.02, 1.42] 66.1% | 0.4 [-1.23, 2.02] 28.6% |

The estimates and the probabilities (%) are for the Interventions (in first column) vs. Comparators (in the second, third, etc. columns).

Probability (%) for the comparison of two specific treatments (not to be confused with the rank probability) indicates the chance that an intervention is better than the comparator treatment.

Abbreviations: CrI = credible interval; MD = mean difference

### Appendix Table 6. Acute Post-operative Bleeding: Rank Probabilities and SUCRA of Each Treatment (%)

| **Treatment** | **Rank 1** | **Rank 2** | **Rank 3** | **SUCRA** |
| --- | --- | --- | --- | --- |
| High | 79.5 | 11.2 | 9.3 | 85.1 |
| Low | 14.4 | 69.3 | 16.3 | 49.0 |
| Standard | 6.2 | 19.4 | 74.4 | 15.9 |

Lower value of the estimate was considered better while ranking treatments.

Abbreviation: SUCRA = surface under the cumulative ranking

### Appendix Table 7. Acute Post-operative Bleeding: Estimates and 95% CrIs of OR and Probabilities for Each Comparison

| **Intervention** | **Comparator Standard**  **OR [95% CrI] %** | **Low**  **OR [95% CrI] %** |
| --- | --- | --- |
| Low | 0.63 [0.18, 1.92] 79.8% |  |
| High | 0.27 [0.02, 2.18] 88.4% | 0.44 [0.06, 2.58] 81.8% |

The estimates and the probabilities (%) are for the Interventions (in first column) vs. Comparators (in the second, third, etc. columns).

Probability (%) for the comparison of two specific treatments (not to be confused with the rank probability) indicates the chance that an intervention is better than the comparator treatment.

Abbreviations: CrI = credible interval; OR = odds ratio

### Appendix Table 8. Post-operative Nausea/Vomiting: Rank Probabilities and SUCRA of Each Treatment (%)

| **Treatment** | **Rank 1** | **Rank 2** | **Rank 3** | **SUCRA** |
| --- | --- | --- | --- | --- |
| Low | 79.2 | 20.2 | 0.6 | 89.3 |
| High | 17.6 | 31.4 | 51.0 | 33.3 |
| Standard | 3.2 | 48.4 | 48.4 | 27.4 |

Lower value of the estimate was considered better while ranking treatments.

Abbreviation: SUCRA = surface under the cumulative ranking

### Appendix Table 9. Post-operative Nausea/Vomiting: Estimates and 95% CrIs of OR and Probabilities for Each Comparison

| **Intervention** | **Comparator Standard**  **OR [95% CrI] %** | **Low**  **OR [95% CrI] %** |
| --- | --- | --- |
| Low | 0.45 [0.17, 1.08] 96.3% |  |
| High | 1.03 [0.14, 10.08] 48.9% | 2.31 [0.39, 19.11] 17.8% |

The estimates and the probabilities (%) are for the Interventions (in first column) vs. Comparators (in the second, third, etc. columns).

Probability (%) for the comparison of two specific treatments (not to be confused with the rank probability) indicates the chance that an intervention is better than the comparator treatment.

Abbreviations: CrI = credible interval; OR = odds ratio

### Appendix Table 10. Duration of Surgery: Rank Probabilities and SUCRA of Each Treatment (%)

| **Treatment** | **Rank 1** | **Rank 2** | **Rank 3** | **SUCRA** |
| --- | --- | --- | --- | --- |
| High | 78.0 | 12.6 | 9.4 | 84.3 |
| Standard | 21.2 | 72.5 | 6.3 | 57.5 |
| Low | 0.7 | 14.9 | 84.3 | 8.2 |

Lower value of the estimate was considered better while ranking treatments.

Abbreviation: SUCRA = surface under the cumulative ranking

### Appendix Table 11. Duration of Surgery: Estimates and 95% CrIs of MD and Probabilities for Each Comparison

| **Intervention** | **Comparator Standard**  **MD [95% CrI] %** | **Low**  **MD [95% CrI] %** |
| --- | --- | --- |
| Low | 1.94 [–0.76, 4.05] 6.8% |  |
| High | –3.08 [–11.26, 4.8] 78.2% | –5.02 [–12.71, 2.64] 90.4% |

The estimates and the probabilities (%) are for the Interventions (in first column) vs. Comparators (in the second, third, etc. columns).

Probability (%) for the comparison of two specific treatments (not to be confused with the rank probability) indicates the chance that an intervention is better than the comparator treatment.

Abbreviations: CrI = credible interval; MD = mean difference

## PRISMA NMA Checklist of Items to Include when Reporting a Systematic Review Involving an NMA

| **Section/Topic** | **Item #** | **Checklist Item** | **Reported on Page #** |
| --- | --- | --- | --- |
| **Title** | | | |
| Title | 1 | Identify the report as a systematic review incorporating a network meta-analysis (or related form of meta-analysis). | 1 |
| **Abstract** | | | |
| Structured summary | 2 | Provide a structured summary including, as applicable:   - Background: main objectives - Methods: data sources; study eligibility criteria, participants, and interventions; study appraisal; and synthesis methods, such as network meta-analysis. - Results: number of studies and participants identified; summary estimates with corresponding confidence/credible intervals; treatment rankings may also be discussed. Authors may choose to summarize pairwise comparisons against a chosen treatment included in their analyses for brevity. - Discussion/Conclusions: limitations; conclusions and implications of findings. - Other: primary source of funding; systematic review registration number with registry name. | 3  Data sources and study appraisal details are reported in pages 7 and 8, due to limited word count in the abstract |
| **Introduction** | | | |
| Rationale | 3 | Describe the rationale for the review in the context of what is already known, including mention of why a network meta-analysis has been conducted. | 6 |
| Objective | 4 | Provide an explicit statement of questions being addressed, with reference to PICOS | 6 |
| **Methods** | | | |
| Protocol and registration | 5 | Indicate whether a review protocol exists and if and where it can be accessed (e.g., Web address); and, if available, provide registration information, including registration number. | A review protocol has been developed but has not been registered and is not publicly available. |
| Eligibility criteria | 6 | Specify study characteristics (e.g., PICOS, length of follow-up) and report characteristics (e.g., years considered, language, publication status) used as criteria for eligibility, giving rationale. Clearly describe eligible treatments included in the treatment network, and note whether any have been clustered or merged into the same node (with justification). | 6 and 7 |
| Information sources | 7 | Describe all information sources (e.g., databases with dates of coverage, contact with study authors to identify additional studies) in the search and date last searched. | 7 |
| Search | 8 | Present full electronic search strategy for at least one database, including any limits used, such that it could be repeated. | Supplemental material |
| Study selection | 9 | State the process for selecting studies (i.e., screening, eligibility, included in systematic review, and, if applicable,  included in the meta-analysis). | 8 |
| Data collection process | 10 | Describe method of data extraction from reports (e.g., piloted forms, independently, in duplicate) and any processes for obtaining and confirming data from investigators. | 8 |
| Data items | 11 | List and define all variables for which data were sought (e.g., PICOS, funding sources) and any assumptions and simplifications made. | 8 |
| Geometry of the network | **S1** | Describe methods used to explore the geometry of the treatment network under study and potential biases related to it. This should include how the evidence base has been graphically summarized for presentation, and what characteristics were compiled and used to describe the evidence base to readers. | 9 and 10 |
| Risk of bias within individual studies | 12 | Describe methods used for assessing risk of bias of individual studies (including specification of whether this was done at the study or outcome level), and how this information is to be used in any data synthesis. | 8 |
| Summary measures | 13 | State the principal summary measures (e.g., risk ratio, difference in means). *Also describe the use of additional summary measures assessed, such as treatment rankings and surface under the cumulative ranking curve values, as well as modified approaches used to present summary findings from meta-analyses.* | 9 |
| Planned methods of analysis | 14 | Describe the methods of handling data and combining results of studies for each network meta-analysis. This should include, but not be limited to:   - *Handling of multi-arm trials* - *Selection of variance structure* - *Selection of prior distributions in Bayesian analyses* - *Assessment of model fit* | 8 and 9 |
| Assessment of consistency | **S2** | Describe the statistical methods used to evaluate the agreement of direct and indirect evidence in the treatment network(s) studied. Describe efforts taken to address its presence when found. | 15 |
| Risk of bias across studies | 15 | Specify any assessment of risk of bias that may affect the cumulative evidence (e.g., publication bias, selective reporting within studies). | 10 and Figure 3 |
| Additional analyses | 16 | Describe methods of additional analyses if done, indicating which were pre-specified. This may include, but not be limited to, the following:   - Sensitivity or subgroup analyses - Meta-regression analyses - Alternative formulations of the treatment network - *Use of alternative prior distributions for Bayesian analyses (if applicable)* | Not applicable |
| Study selection | 17 | Give numbers of studies screened, assessed for eligibility, and included in the review, with reasons for exclusions at each stage, ideally with a flow diagram. | Figure 2 |
| Presentation of network structure | **S3** | Provide a network graph of the included studies to enable visualization of the geometry of the treatment network. | Figure 1 |
| Summary of network geometry | **S4** | Provide a brief overview of characteristics of the treatment network. This may include commentary on the abundance of trials and randomized patients for the different interventions and pairwise comparisons in the network, gaps of evidence in the treatment network, and potential biases reflected by the network structure. | 8 |
| Study characteristics | 18 | For each study, present characteristics for which data were extracted (e.g., study size, PICOS, follow-up period) and provide the citations. | Table 1 |
| Risk of bias within studies | 19 | Present data on risk of bias of each study and, if available, any outcome level assessment. | Figure 3 |
| Results of individual studies | 20 | For all outcomes considered (benefits or harms), present, for each study: 1) simple summary data for each intervention group, and 2) effect estimates and confidence intervals. *Modified approaches may be needed to deal with information from larger networks.* | Figures 4, 5, and 6 |
| Synthesis of results | 21 | Present results of each meta-analysis done, including confidence/credible intervals. *In larger networks, authors may focus on comparisons versus a particular comparator (e.g., placebo or standard care), with full findings presented in an appendix. League tables and forest plots may be considered to summarize pairwise comparisons.* If additional summary measures were explored (such as treatment rankings), these should also be presented. | 10, 11, and 12  Figures 4, 5, and 6  Supplementary material |
| Exploration for inconsistency | **S5** | Describe results from investigations of inconsistency. This may include such information as measures of model fit to compare consistency and inconsistency models, *P* values from statistical tests, or summary of inconsistency estimates from different parts of the treatment network. | Not applicable |
| Risk of bias across studies | 22 | Present results of any assessment of risk of bias across studies for the evidence base being studied. |  |
| Results of additional analyses | 23 | Give results of additional analyses, if done (e.g., sensitivity or subgroup analyses, meta-regression analyses*, alternative network geometries studied, alternative choice of prior distributions for Bayesian analyses,* and so forth). | Not applicable |
| **Discussion** | | | |
| Summary of evidence | 24 | Summarize the main findings, including the strength of evidence for each main outcome; consider their relevance to key groups (e.g., healthcare providers, users, and policy-makers). | 12 and 13 |
| Limitations | 25 | Discuss limitations at study and outcome level (e.g., risk of bias), and at review level (e.g., incomplete retrieval of identified research, reporting bias). *Comment on the validity of the assumptions, such as transitivity and consistency. Comment on any concerns regarding network geometry (e.g., avoidance of certain comparisons).* | 15 and 16 |
| Conclusions | 26 | Provide a general interpretation of the results in the context of other evidence, and implications for future research. | 16 |
| **Funding** | | | |
| Funding | 27 | Describe sources of funding for the systematic review and other support (e.g., supply of data); role of funders for the systematic review. This should also include information regarding whether funding has been received from manufacturers of treatments in the network and/or whether some of the authors are content experts with professional conflicts of interest that could affect use of treatments in the network. | 1 |

* Text in italics indicates wording specific to reporting of network meta-analyses that has been added to guidance from the PRISMA statement.

† Authors may wish to plan for use of appendices to present all relevant information in full detail for items in this section.

Abbreviation: PICOS = population, intervention/comparators, outcomes, study design.

## GRADE Assessment

This section provides a summary of the confidence in estimates produced by our network meta-analysis (NMA). We rated the evidence according to each domain of the Grading of Recommendations Assessment, Development, and Evaluation (GRADE) assessment tool.^[[1]](#footnote-1)^

**Indirectness:** In our network, when all three intra-abdominal pressure (IAP) levels were in an analysis, no included studies directly compared high vs. standard IAP. Since low IAP was a common comparator connecting standard and high IAP, this allowed estimation of the relative effects of the three IAP levels, including high vs. standard IAP. However, the lack of head-to-head trials raises the possibility of indirectness (in this case, reliance on indirect [i.e., lower quality] evidence) for the NMA estimates in question. It is also important to note that, while the target population in the included trials was patients undergoing laparoscopic cholecystectomy, the recruited individuals might not have been typical of those undergoing such an intervention in real-world settings. Given these two potential sources of indirectness, we have assigned moderate confidence to all estimates for this domain.

**Intransitivity:** To minimize possible heterogeneity and improve the transitivity (i.e., similarity of patient/study effect-modifying characteristics across trials), the NMA was limited to studies that analyzed outcomes for patients undergoing laparoscopic cholecystectomy. However, intransitivity was possible in a network where all three IAP levels were compared, given that patients could have differed across trials with respect to known and unknown effect modifiers. Therefore, the conclusiveness of evidence on this domain was deemed moderate due to potential intransitivity.

**Incoherence** (sometimes called “inconsistency” in NMA literature): Incoherence (i.e., disparity between direct and indirect effect estimates for a given comparison) is generally caused by intransitivity, indirectness, and/or biases (e.g., publication bias). However, there was no issue of incoherence in our NMA because no comparisons in evidence network had direct and indirect evidence available. There was no reason to reduce the confidence in our estimates because of incoherence.

**Inconsistency** (sometimes called heterogeneity): The relative effects for low vs. high IAP across trials were highly inconsistent or heterogeneous for some outcomes; this was reflected in the high statistical heterogeneity of the related NMA. This reduced the certainty of those estimates, and we have assigned them low confidence. For example, evidence on mean duration of surgery with high vs. low IAP was highly heterogeneous and in opposite directions across studies; some studies concluded that high IAP led to a shorter duration of surgery, and some studies indicated that administrating low IAP levels reduced the surgery time. Accordingly, the Bayesian NMA estimate for this comparison was MD: –5.02 (–12.71 to 2.64) minutes, suggesting that high IAP was associated with an operating time that is five minutes shorter—this result was very uncertain (given the wide credible interval around the point estimate).

**Imprecision:** The confidence intervals within included studies and the confidence or credible intervals in the effects from meta-analysis or NMA were generally wide—only one or two studies with small sizes were available for most networks. Also, the event rates (where applicable) were low in high vs. standard comparisons, which had no direct evidence. For example, for conversion to open surgery, there were few events (conversions) in included studies and the estimate (95% credible interval) of the odds ratio of conversion in low vs. standard IAP was 2.73 (0.87 to 10.75). While this estimate suggests high risk of conversion in the low IAP group, it is important to recognize that this result is imprecise and might change if sufficiently large studies are conducted in the future and are used to inform subsequent NMAs. Similarly, the NMA provided imprecise estimates of the comparative effects of high vs. low IAP on the mean duration of surgery, meaning that there is low confidence in the results, as they may change if new, larger studies become available.

**Risk of bias:** Based on assessment with the Cochrane Risk of Bias tool,^[[2]](#footnote-2)^ 68% of the included trials had high overall risk of bias. Approximately 64% and 27% of the studies had moderate and high risk of selection bias, respectively. Confidence in the estimates for all outcomes must be rated as moderate to low due to potential high risk of biases.

**Publication bias:** There may be publication bias in our review, as the included studies in were small or moderately sized (with study samples ranging from 18 to 148 patients). For example, some studies with small sizes might not have been submitted for publication or published due to lack of statistically significant results. In view of these risks, we have assigned moderate-to-low confidence to our NMA results for different outcomes.

**Overall confidence:** We assigned very low-to-low certainty to the estimates of effects for most outcomes analyzed in our NMA, because in all but a few outcomes, confidence in the NMA results was undermined by one or more issues (e.g., imprecision or heterogeneity). This means the estimates of effects for some outcomes might easily change when data from large, well-conducted trial are available for inclusion in future NMAs.

1. Salanti G, Del Giovane C, Chaimani A, Caldwell DM, Higgins JP. Evaluating the quality of evidence from a network meta-analysis. PLoS One. 2014;9(7):e99682. [↑](#footnote-ref-1)
2. Higgins JP, Altman DG, Gotzsche PC, et al. The Cochrane Collaboration's tool for assessing risk of bias in randomised trials. BMJ. 2011;343:d5928. [↑](#footnote-ref-2)
